# Vitamin K2 Promotes Mitochondrial Structural and Functional Homeostasis to Ameliorate Alzheimer Pathology by Targeting the EGFR-Ras-ERK Signaling Axis

**DOI:** 10.3390/ijms27135708

**Published:** 2026-06-24

**Authors:** Yanan Li, Hanyu Zhao, Jie Wu, Yan Hu, Juhong Pan, Asante Obed Frimpong, Biguo Xie, Wanming Yang, Manman Sun, Wenjun Chen, Peng Wang, Changsheng Shao

**Affiliations:** 1 Department of Nutrition and Food Hygiene, School of Public Health, Anhui Medical University, Hefei 230032, China; lyn990716@163.com (Y.L.); zhaohanyu0210@163.com (H.Z.); 13167849788@163.com (J.W.); 19155363615@163.com (Y.H.); panjh2025@163.com (J.P.); xie_biguo@163.com (B.X.); ywm200112@163.com (W.Y.); 2 Key Laboratory of High Magnetic Field and Ion Beam Physical Biology, Hefei Institutes of Physical Science, Chinese Academy of Sciences, Hefei 230026, China; ofrimpong28@yahoo.com (A.O.F.); mmsun@iim.ac.cn (M.S.); shaochangsheng@hmfl.ac.cn (C.S.)

**Keywords:** Vitamin K2, Alzheimer’s disease, network pharmacology, molecular docking, *Drosophila melanogaster*, EGFR-Ras-ERK signaling pathway

## Abstract

Alzheimer’s disease (AD) is a progressive neurodegenerative disorder characterized by β-amyloid (Aβ) accumulation and a breakdown of mitochondrial homeostasis. Vitamin K2 (VK2) has emerged as a potential neuroprotective agent, yet the specific molecular cascades linking its intervention to the restoration of mitochondrial integrity remain poorly understood. This study utilizes an AD *Drosophila* model to investigate the efficacy of VK2 and elucidates its multidimensional regulatory mechanisms. Behavioral analysis showed that VK2 significantly rescued locomotor impairments, improving both vertical climbing and horizontal walking performance. Crucially, VK2 intervention achieved a systemic rescue of mitochondrial health: transmission electron microscopy (TEM) confirmed the preservation of mitochondrial ultrastructure and cristae density, while biochemical assays demonstrated a robust recovery of bioenergetic markers, including ATP levels and the NAD^+^/NADH ratio. Furthermore, VK2 treatment stabilized the mitochondrial membrane potential (MMP) and effectively attenuated the accumulation of reactive oxygen species (ROS). To identify the molecular drivers of this recovery, an unbiased integration of human clinical transcriptomic data and network pharmacology prioritized the EGFR-Ras-ERK signaling axis as a central hub. In vivo validation confirmed that VK2 suppresses the pathological overactivation of this cascade. VK2 reduced EGFR phosphorylation in parallel with the effects observed for the EGFR inhibitor Gefitinib. Collectively, our findings show that VK2 ameliorates locomotor deficits and mitochondrial dysfunction in Aβ42-expressing flies and that these effects are associated with suppression of the EGFR-Ras-ERK signaling axis. Further studies are required to establish direct target engagement and pathway causality.

## 1. Introduction

Alzheimer’s disease (AD) is a neurodegenerative disorder characterized by an insidious onset and multidimensional cognitive impairments, including memory, learning and language deficits, ultimately compromising daily living activities [1]. Globally, dementia affects over 50 million individuals, with annual costs exceeding US$1 trillion; in China alone, AD and related dementias impact 13.14 million people, representing ~25.5% of the worldwide burden and imposing substantial socioeconomic strain [2]. AD pathogenesis involves multifaceted processes, such as β-amyloid (Aβ) aggregation, tau hyperphosphorylation, neuroinflammation and oxidative stress [3,4].

Beyond proteopathic changes, mitochondrial dysfunction has emerged as a pivotal therapeutic target in AD [5]. Mitochondrial quality control (MQC)—encompassing biogenesis, dynamics and mitophagy—maintains cellular homeostasis and preserves organelle integrity [6]. In AD, MQC disruption leads to mitochondrial accumulation, oxidative stress and bioenergetic failure, exacerbating neurodegeneration [7]. Thus, restoring mitochondrial homeostasis, particularly the integrity of mitochondrial cristae and energy output, offers a promising strategy for slowing disease progression.

Current AD therapies, including Aβ-targeted agents like lecanemab, primarily provide symptomatic relief and fail to halt underlying pathology, highlighting the need for multi-target strategies [8,9,10]. Vitamin K2 (VK2), a naphthoquinone class with anticoagulant properties, exhibits neuroprotective potential in AD [11]. VK2 (including menaquinone-4) is primarily derived from gut microbiota or fermented foods. Recent studies using stable isotope tracing in rodent models have demonstrated that phylloquinone from vegetables can be converted into menaquinone-4 in various tissues in vivo, and is capable of crossing the blood–brain barrier to accumulate in the brain. Consequently, VK2 accumulates in brain tissue, where it alleviates oxidative stress, neuroinflammation, mitochondrial dysfunction, and Aβ-induced neuronal damage [12,13,14,15]. While VK2 has been reported to influence mitochondrial electron transfer and reduce neuroinflammation, the precise molecular cascades that bridge extracellular signaling regulation and the comprehensive restoration of mitochondrial homeostasis in AD remain poorly understood.

To elucidate these mechanisms, we employed an unbiased integration of network pharmacology and human clinical transcriptomic analysis to predict compound target pathway networks [16]. This data-driven approach was complemented by the Aβ42-transgenic *Drosophila melanogaster* model, which features a short lifecycle and conserved neurobiology, enabling efficient in vivo validation of neuroprotective mechanisms [17,18].

Here, we combine systems-level bioinformatics with *Drosophila melanogaster* to delineate the anti-AD mechanisms of VK2. Behavioral assays revealed that VK2 protects against locomotor deficits in AD flies, while cellular analyses demonstrated the systemic rescue of mitochondrial morphology, membrane potential, and energy metabolism. Crucially, we identify the EGFR-Ras-ERK signaling axis as a central hub through which VK2 exerts its neuroprotective effects. Our findings show that VK2 suppresses the pathological overactivation of this axis to preserve mitochondrial structural and functional integrity, providing a cohesive mechanistic framework that supports the potential of VK2 as a promising nutritional intervention for Alzheimer’s disease.

## 2. Results

### 2.1. Vitamin K2 Improves Locomotor Deficits in an AD Drosophila Model

**Figure 1:**
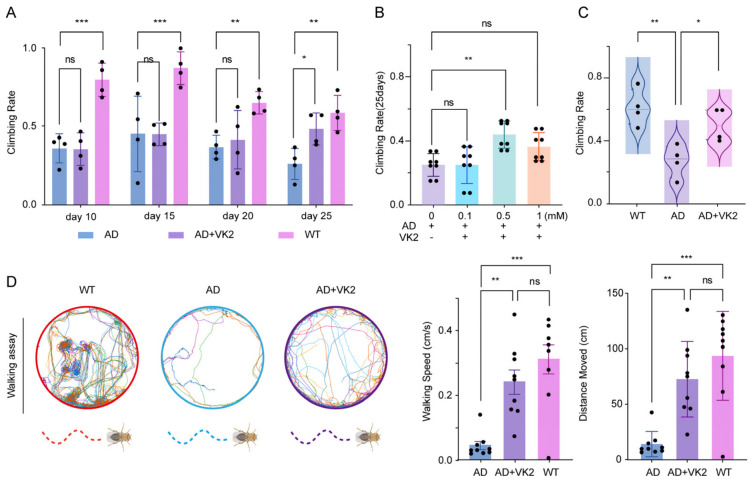
Vitamin K2 improves climbing and locomotor performance in an AD *Drosophila* model. (**A**–**C**) Effects of VK2 on climbing ability. (**A**) Climbing performance over time in AD, AD + VK2 (0.5 mM), and WT groups. AD flies show a progressive decline, which is significantly rescued by VK2. (**B**) Climbing performance at different VK2 concentrations (0, 0.1, 0.5, 1 mM) measured at day 25. (**C**) Climbing performance on day 25. (**D**) Effects on walking speed. Representative walking trajectories from each treatment group are shown (left; WT, AD, AD + VK2 colored pink, blue, and purple, respectively). Walking speed (cm/s) for AD, AD + VK2, and WT groups is depicted in the middle; distance moved is shown on the right. WT group: elav-W1118; AD group: elav-Aβ42; AD + VK2 group: elav-Aβ42 + 0.5 mM VK2. Data information: Data are presented as mean ± standard error of mean (SEM). *n* = 100 biological replicates per group. Panel **A** was analyzed by Two-way ANOVA, Panels **B**–**D** by One-Way ANOVA. Statistical annotation: ns, *p* > 0.05; * *p* < 0.05; ** *p* < 0.01; *** *p* < 0.001.

To comprehensively evaluate the locomotor performance of Aβ42-transgenic *Drosophila*, we conducted both climbing and walking tracking assays to assess movement across different spatial axes. Specifically, the climbing assay was used to measure negative geotaxis (vertical movement), while the walking tracking assay quantified spontaneous locomotion in a planar arena (horizontal movement). We first performed a longitudinal assessment of climbing ability at days 10, 15, 20, and 25. AD-model flies exhibited a progressive decline in vertical locomotor function as the disease progressed. While the groups showed similar performance in the early stages (days 10–20), a statistically significant divergence between the untreated AD group and the VK2-treated group emerged at day 25 (*p* < 0.05; Figure 1A). Quantitatively, the climbing index of AD flies at day 25 was significantly lower than that of age-matched controls (0.26 ± 0.10 vs. 0.59 ± 0.11; *n* = 100, *p* < 0.05), but was significantly rescued by VK2 intervention (0.48 ± 0.10; *p* < 0.01).

To identify the optimal therapeutic dose for these locomotor improvements, we evaluated the efficacy of VK2 across a concentration gradient (0.1, 0.5, and 1.0 mM). At the 25-day mark, the 0.1 mM dose failed to produce a significant improvement relative to the untreated AD group (*p* > 0.05; Figure 1B). In contrast, both 0.5 mM and 1.0 mM concentrations resulted in highly significant increases in climbing rates (*p* < 0.01). Because the 1.0 mM concentration did not yield a further significant gain in performance compared to 0.5 mM, we selected 0.5 mM as the optimized dose for all subsequent mechanistic experiments. The rescue effect of this optimized dose was confirmed in a large cohort at day 25 (Figure 1C). These findings were further corroborated by horizontal walking trajectory tracking, which showed that 0.5 mM VK2-treated flies regained walking speeds (0.24 ± 0.03 cm/s vs. 0.04 ± 0.01 cm/s in AD; *p* < 0.01) and total travel distances that were otherwise lost due to Aβ42 toxicity (Figure 1D). Thus, VK2 mitigates motor dysfunction in the AD *Drosophila* model, highlighting its neuroprotective potential.

### 2.2. VK2 Restores Mitochondrial Ultrastructural Integrity and Bioenergetic Homeostasis

**Figure 2:**
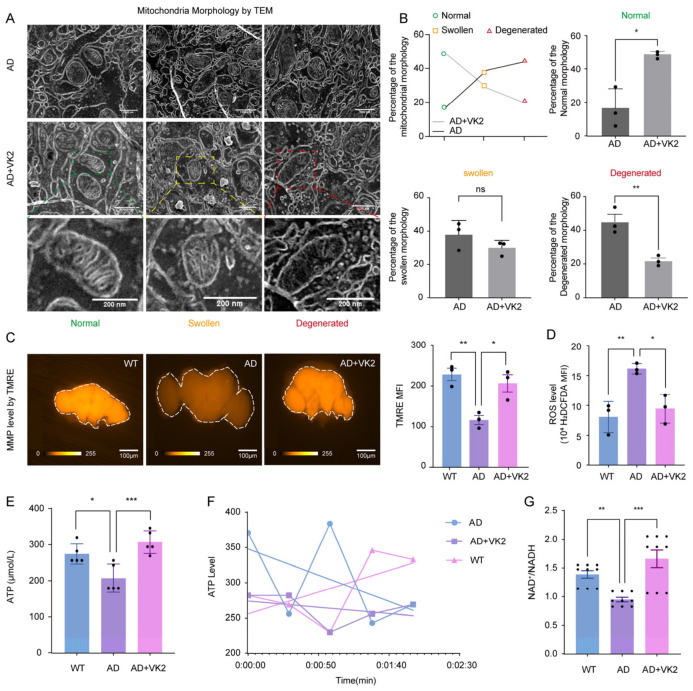
VK2 modulates mitochondrial function in AD flies. (**A**) Mitochondrial morphology in AD and AD + VK2 brains assessed by TEM. Mitochondria categorized as normal (organized cristae, green), swollen (cristae swollen, yellow), or degenerated (cristae lost, red). Scale bars: 1000 nm (top) and 200 nm (bottom, magnified 5×). (**B**) Quantification of mitochondrial damage in brains after VK2 treatment (For each condition, analyses were performed using three brains, with a minimum of 30 mitochondria examined per sample). Left: percentage of mitochondria by morphology; top-right: percentage of normal mitochondria; bottom-left: percentage of swollen mitochondria; bottom-right: percentage of degenerated mitochondria. (**C**) Mitochondrial membrane potential in freshly dissected adult fly brains assessed by TMRE staining under live-tissue conditions. Representative fluorescence images (**left**) and quantification of TMRE intensity (**right**) are shown. Scale bar = 100 μm. (**D**) Intracellular reactive oxygen species (ROS) levels in freshly dissected, unfixed adult fly brains assessed by H_2_DCFDA staining under live-tissue conditions. Quantification of H_2_DCFDA fluorescence intensity is shown. (**E**,**F**) Determination of ATP levels and ATP kinetic levels. (**G**) Determination of NAD^+^/NADH ratio in each group. WT group: elav-W1118; AD group: elav-Aβ42; AD + VK2 group: elav-Aβ42 + 0.5 mM VK2. Data information: Data are presented as mean ± standard error of mean (SEM). Panel **B** was analyzed by *t*-test (*n* = 3 biological replicates per group), Panel **C** by One-Way ANOVA (*n* = 5 biological replicates per group), Panels **D**–**G** by One-Way ANOVA followed by Tukey’s post hoc test (*n* = 40 biological replicates per group). Statistical annotation: ns, *p* > 0.05; * *p* < 0.05; ** *p* < 0.01; *** *p* < 0.001.

Given that these locomotor improvements likely depend on the energy state of brain cells, we investigated whether the optimized dose (0.5 mM) of VK2 influences mitochondrial architecture and function. Transmission electron microscopy (TEM) of brain tissues revealed distinct ultrastructural pathologies in AD flies, including mitochondrial swelling and a significant loss of cristae density—the physical scaffolding for oxidative phosphorylation (Figure 2A). Quantitative analysis demonstrated that VK2 intervention significantly attenuated these structural impairments, with the proportion of morphologically intact mitochondria increasing from 16.7 ± 12.0% to 48.8 ± 2.2% (*n* = 3 brains per group, *p* < 0.05; Figure 2B).

To determine if this structural restoration translated into functional bioenergetic gains, we first measured the mitochondrial membrane potential and oxidative stress levels. Intervention with 0.5 mM VK2 significantly elevated the MMP from 116 ± 19 to 206 ± 37 arbitrary units (Figure 2C), accompanied by a 41.3% reduction in reactive ROS levels (*p* < 0.05; Figure 2D). Furthermore, we quantified the steady-state levels of ATP and the NAD^+^/NADH ratio to evaluate the overall metabolic flux and energy output of the mitochondrial machinery. ATP serves as the primary energy currency for neuronal survival and synaptic transmission, both of which are compromised in AD. AD-model flies also showed reduced ATP content compared with WT controls (207 ± 39 vs. 274 ± 28 μmol/L), whereas VK2 significantly restored ATP levels (Figure 2E). Kinetic analysis further revealed that VK2-treated flies maintained more stable ATP levels over time, avoiding the progressive metabolic exhaustion observed in untreated AD flies (Figure 2F). In parallel, we measured the NAD^+^/NADH ratio to assess the redox state and efficiency of the electron transport chain. The NAD^+^/NADH ratio is a critical indicator of mitochondrial metabolic health and a key cofactor for NAD^+^-dependent protective enzymes. In AD flies, this ratio was significantly reduced to 0.95 ± 0.12, indicating a state of metabolic dysfunction. Treatment with 0.5 mM VK2 significantly elevated the NAD^+^/NADH ratio to 1.66 ± 0.46 (*p* < 0.001; Figure 2G), suggesting enhanced oxidative phosphorylation efficiency and restored redox balance. Collectively, these data indicate that 0.5 mM VK2 does not merely preserve mitochondrial appearance but actively maintains the bioenergetic flux necessary for functional recovery.

### 2.3. Network Pharmacology and Molecular Docking Identify the EGFR-Ras-ERK Axis as a Potential Mechanistic Target

**Figure 3:**
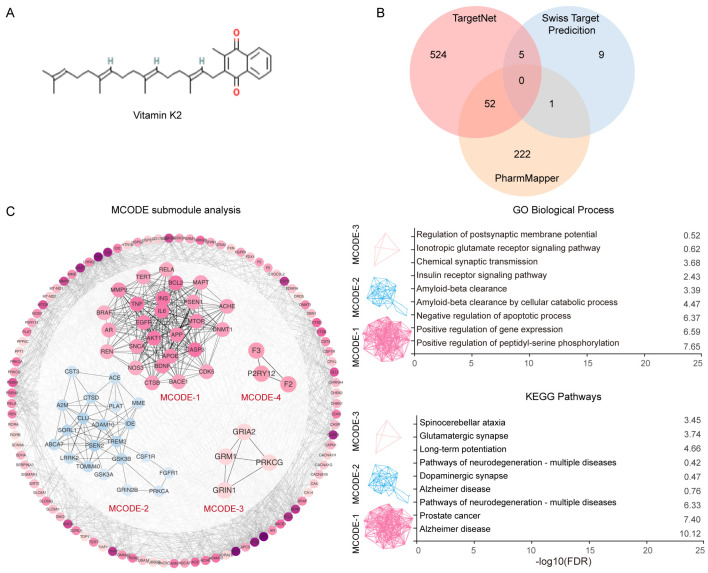
VK2–responsive target genes and network analysis. (**A**) Chemical structure of Vitamin K2. (**B**) Intersections between VK2 target genes. Targets were retrieved from TargetNet, Swiss Target Prediction, and PharmMapper. Intersection sizes: TargetNet∩Swiss Target Prediction = 5; TargetNet∩PharmMapper = 52; PharmMapper∩Swiss Target Prediction = 1; no triple intersection. (**C**) PPI network and MCODE submodules. Identification of 118 VK2-related neurological disease targets from 746 VK2-associated targets and construction of their PPI network. Key clusters identified by MCODE include Cluster 1–3. Pink color intensity represents the degree value, with darker colors indicating higher connectivity. GO enrichment for the top three clusters highlights positively regulated phosphotyrosine signaling, negative regulation of apoptosis, and positive regulation of gene expression. KEGG enrichment for these clusters identifies pathways linked to Alzheimer’s disease and neurodegenerative processes. The x-axis represents −log10(FDR), where larger values indicate greater enrichment significance.

To investigate the molecular signaling network through which VK2 exerts its effects, an integrative network pharmacology framework was employed. Potential targets of VK2 (menaquinone-4, MW: 446.71 Da) were identified by integrating literature mining with chemogenomic databases, yielding a set of 746 unique candidates (Figure 3A,B). Protein–protein interaction (PPI) network analysis was performed to evaluate the functional connectivity of these targets, resulting in an interactome with a clustering coefficient of 0.452 (Figure 3C). Functional clustering via Molecular Complex Detection (MCODE) identified modules associated with the regulation of apoptosis and Aβ clearance, indicating that VK2 interacts with regulatory nodes relevant to AD pathology.

**Figure 4:**
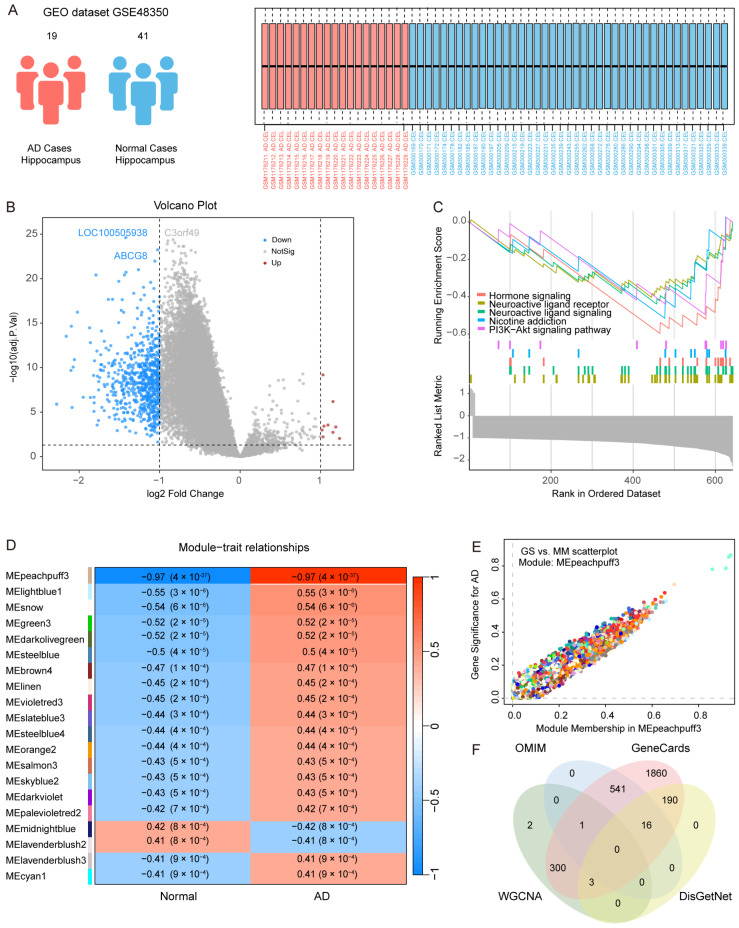
AD-related gene screen analysis. (**A**) DEG-based AD gene set from the GEO dataset GSE48350 comprising 41 control and 19 AD hippocampal samples; sample IDs are shown on the right. (**B**) Volcano plot of DEGs between AD and control. Upregulated genes are red, downregulated are blue, non-significant are gray. (**C**) GSEA for GSE48350 showing enriched pathways among upregulated and downregulated genes. (**D**) WGCNA of normal vs. AD samples displaying module-trait relationships; each cell shows the correlation coefficient (*p* value). Positive correlations (red); Negative correlation (blue). Color intensity reflecting the correlation strength. (**E**) Correlation between MEpeachpuff3 module genes and AD traits. (**F**) Intersection analysis of AD-target genes across WGCNA-identified modules with GeneCards, OMIM, and DisGeNET; Venn diagram illustrating overlaps.

**Figure 5:**
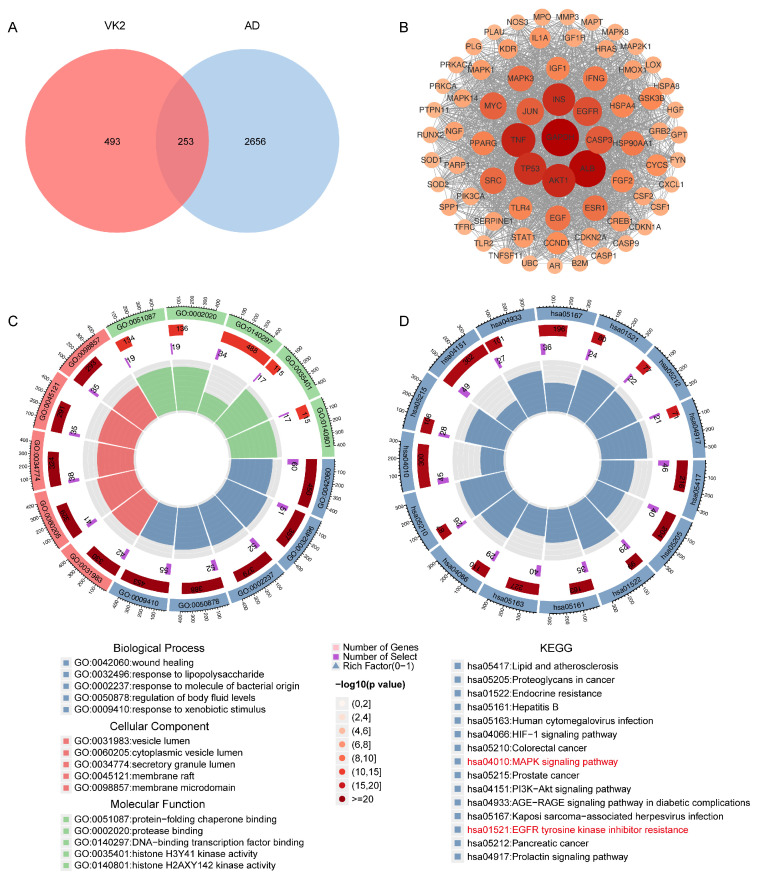
Integration and analysis of intersecting target genes between VK2 and Alzheimer’s disease. (**A**) Venn diagram of the intersecting target genes between VK2 and AD. The intersection identifies 253 shared genes potentially relevant for AD therapy. (**B**) Protein–protein interaction (PPI) network of the 77 intersecting genes. The 77 core targets with high connectivity (degree > 65) in the 253 targets are highlighted by larger node size and deeper color, which is closer to the network center. (**C**) Gene Ontology (GO) enrichment analysis of core intersecting targets. The innermost ring shows enriched GO terms by biological category; red intensity in the second ring reflects statistical significance (represented by −log10 P-adjusted value, with an FDR < 0.05 threshold); the third ring depicts gene counts (purple squares); blue triangles indicate Rich Factor. (**D**) KEGG pathway enrichment analysis of these intersecting genes. The outer ring lists enriched pathways; inner rings display statistical significance (red color, −log10 P-adjusted value, FDR < 0.05), gene counts (purple squares), and enrichment (blue triangles). Pathways highlighted in red were the primary focus of this study. The entire annotated gene set of Homo sapiens was used as the enrichment background for GO and KEGG enrichment analyses.

To prioritize these targets within a clinical context, the VK2-responsive interactome was integrated with AD-associated transcriptomic signatures derived from human hippocampal samples (GSE48350). Differentially expressed genes (DEGs) were identified between AD patients and age-matched controls (Figure 4A,B). Weighted Gene Co-expression Network Analysis (WGCNA) partitioned these genes into 17 co-expression modules, among which the ME. peachpuff3 module exhibited a negative correlation with AD diagnosis (*r* = −0.97, *p* = 4 × 10^−37^; Figure 4C–E). Intersection analysis between the VK2-target pool and 2909 curated AD genes yielded 253 overlapping targets (Figure 5A), from which 77 hub genes were identified on the basis of degree centrality > 65 (Figure 5B). KEGG pathway enrichment analysis showed that these hub genes were significantly enriched in the EGFR-Ras-ERK signaling cascade, with network topology identifying EGFR, Ras, Raf, and ERK as the central downstream effectors (Figure 5C,D, Supplement Figure S1). Specifically, Ras was identified as a convergence point, suggesting that VK2 may modulate AD progression through the regulation of the EGFR-Ras-ERK signaling axis.

**Figure 6:**
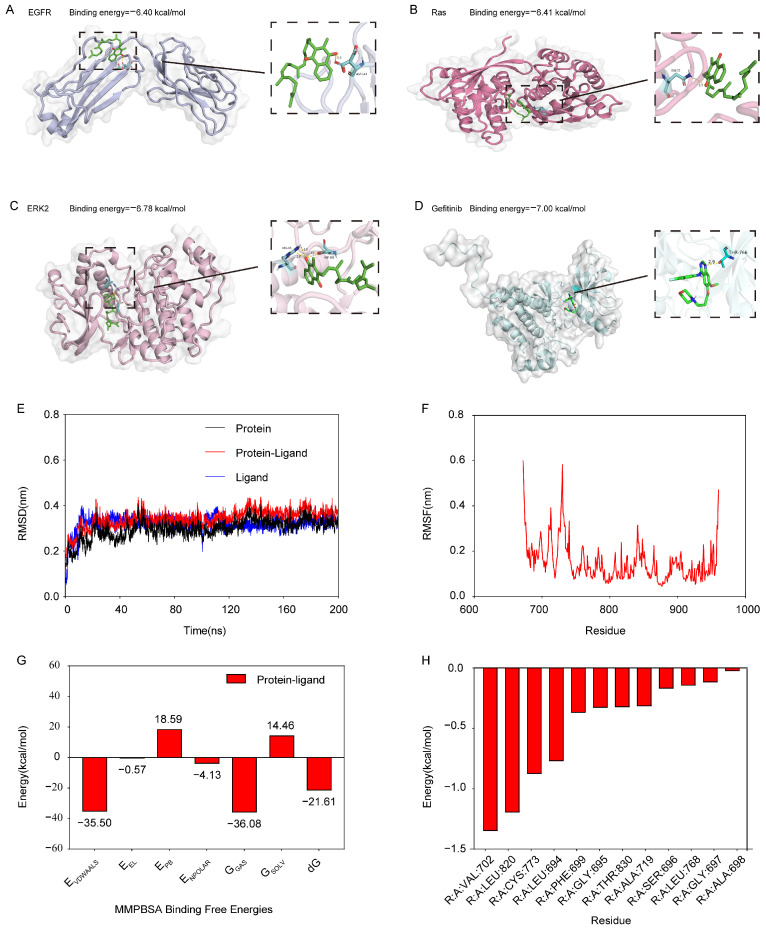
Molecular docking and molecular dynamics simulation of Vitamin K2 with key proteins in the EGFR-Ras-ERK pathway. (**A**) Predicted binding mode of Vitamin K2 (VK2) within the active site of EGFR (PDB: 3POZ), showing stable occupation of the kinase pocket with a calculated binding energy of −6.40 kcal/mol. Enlarged views highlight interactions between VK2 and residues located near the ATP-binding region of the EGFR kinase domain. (**B**) Predicted binding mode of VK2 with Ras (PDB: 5WDS), exhibiting a binding energy of −6.41 kcal/mol and hydrophobic interactions within the active pocket. (**C**) Predicted binding mode of VK2 with ERK (PDB: 5ETC), showing a binding energy of −6.78 kcal/mol and stable localization within the catalytic region of the kinase domain. (**D**) Docking pose of the clinical EGFR inhibitor Gefitinib within the EGFR kinase domain (PDB: 3POZ), with a binding energy of −7.00 kcal/mol. VK2 partially overlapped with the Gefitinib-binding region, particularly near residues associated with the ATP-binding pocket. (**A**–**D**) Ligands are shown in green, proteins as colored ribbon structures, key interacting residues in light blue, and hydrogen bonds as yellow dashed lines. Numbers above the panels indicate binding free energies (kcal/mol). (**E**) RMSD analysis of the VK2–EGFR complex during molecular dynamics simulation. (**F**) RMSF analysis of EGFR residues in the VK2—EGFR complex. (**G**) MM-PBSA binding free-energy analysis of the VK2–EGFR complex. (**H**) Per-residue energy decomposition analysis of the VK2—EGFR complex.

To evaluate the biophysical interaction between VK2 and these kinase hubs, molecular docking simulations were performed to assess binding. The interactions between VK2 and the catalytic pockets of EGFR, ERK, and Ras were examined, with Gefitinib included as a reference ligand for EGFR-targeted signaling. VK2 exhibited binding affinities for the three targets as follows: EGFR (−6.40 kcal/mol), ERK2 (−6.78 kcal/mol), and Ras (−6.41 kcal/mol). In EGFR, VK2 was predicted to interact with residues including Cys773 and Gly772 within the ATP-binding hinge region, together with hydrophobic contacts involving Leu694, Val702, Ala719, and Lys721 located in the catalytic pocket adjacent to the activation-associated region (Figure 6A–C). Notably, the hydrogen-bond interaction with Cys773 is of particular interest because this conserved hinge-region residue is frequently involved in canonical EGFR inhibitor binding. VK2 occupied a region within the EGFR binding pocket that partially overlapped with the Gefitinib-binding region (−7.00 kcal/mol), which occupies the ATP-binding pocket through π-alkyl interactions and electrostatic attraction (Figure 6D). However, these computational docking results should be interpreted cautiously, as they do not establish direct target engagement or ATP-competitive inhibition in vivo. Instead, the findings support EGFR-Ras-ERK signaling as a plausible candidate pathway associated with VK2-mediated neuroprotective effects and warrant further experimental validation.

### 2.4. Molecular Dynamics Simulation and MM-PBSA Evaluation of the VK2–EGFR Complex

To further evaluate the dynamic stability and energetic plausibility of the VK2–EGFR interaction, molecular dynamics (MD) simulations and MM-PBSA binding free-energy analyses were subsequently performed. RMSD trajectories demonstrated that the protein–ligand complex reached a stable conformational equilibrium after approximately 10 ns and remained stable throughout the 200 ns simulation period (Figure 6E). RMSF analysis further indicated relatively limited residue fluctuations within the binding region, supporting the structural stability of the complex under dynamic solvent conditions (Figure 6F). MM-PBSA analysis revealed a favorable total binding free energy (ΔG = −21.61 kcal/mol), with van der Waals interactions representing the dominant energetic contribution to complex stabilization (Figure 6G). Per-residue energy decomposition further identified VAL702, LEU820, CYS773, LEU694, and PHE699 as major contributors to ligand binding (Figure 6H), consistent with the docking-predicted localization of VK2 within the ATP-associated catalytic pocket. Collectively, these computational analyses suggest that VK2 may stably associate with EGFR-related signaling components under dynamic conditions, although these findings remain predictive and do not establish direct target engagement or inhibitory activity in vivo.

### 2.5. Validation in Drosophila Brain Tissues Confirms the Suppression of the EGFR-Ras-ERK Signaling Cascade

**Figure 7:**
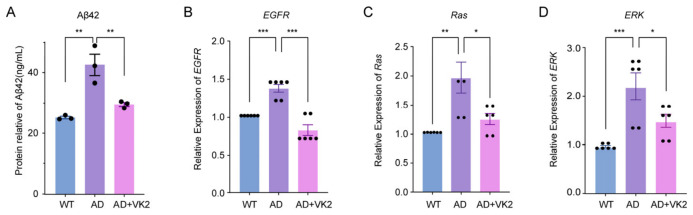
The effect of vitamin K2 on the expression of Aβ42 protein and the mRNA expression of key targets of the EGFR-Ras-ERK pathway in Alzheimer’s disease (AD). (**A**) ELISA was used to investigate the effect of VK2 intervention on the protein level of Aβ42 in the AD model group. (**B**–**D**) RT-qPCR was used to explore the mRNA levels of key targets (*EGFR*, *Ras*, and *ERK*) in the EGFR-Ras-ERK signaling pathway. WT group: elav-W1118; AD group: elav-Aβ42; AD + VK2 group: elav-Aβ42 + 0.5 mM VK2. Data information: Data are presented as mean ± standard error of mean (SEM). Statistical significance was determined by one-way ANOVA followed by Tukey’s post hoc test. Statistical annotation: * *p* < 0.05; ** *p* < 0.01; *** *p* < 0.001.

To validate the computational predictions in a biological system, we first quantified the amyloid levels of the Amyloid-beta 42 (Aβ42) in *Drosophila* brain tissues (Figure 7A). Since Aβ accumulation is the central pathological feature of the AD model, this experiment served to confirm that VK2 intervention addresses the root of the amyloid pathology. AD model flies exhibited a significant upregulation of Aβ42 protein compared to age-matched controls (42.58 ± 6.18 vs. 25.2 ± 0.71; *n* = 50 brains per group, *p* < 0.05). Following 0.5 mM VK2 intervention, Aβ42 protein levels were significantly reduced to 29.6 ± 1.10 (*p* < 0.05 vs. AD group). These data indicate that VK2 reduced Aβ42 protein burden in the fly brain; although the underlying mechanism remains to be determined, these results suggest that VK2 mitigates neurotoxicity at the protein level.

Following the confirmation of reduced Aβ42 expression, we evaluated the mRNA levels of the core components within the EGFR-Ras-ERK signaling cascade to assess the regulatory influence of VK2 (Figure 7B–D). Based on the hub genes identified in the network pharmacology analysis, we focused on the transcriptional modulation of EGFR, Ras, and ERK. In the brain tissues of AD flies, *EGFR* and *Ras* mRNA levels were significantly higher than those in controls, with *EGFR* expression increased by approximately 136% (*p* < 0.001). Treatment with 0.5 mM VK2 successfully lowered *EGFR* mRNA to 0.81 ± 0.17 (*n* = 50 brains per group, *p* < 0.05) and *Ras* mRNA from 1.95 ± 0.65 to 1.24 ± 0.24 (*p* < 0.01). Additionally, *ERK* mRNA expression showed a 26% reduction compared to the untreated AD group (*p* < 0.05). These results indicate that VK2 intervention suppresses the overactive transcriptional landscape of the EGFR-Ras-ERK pathway in the AD fly brain.

**Figure 8:**
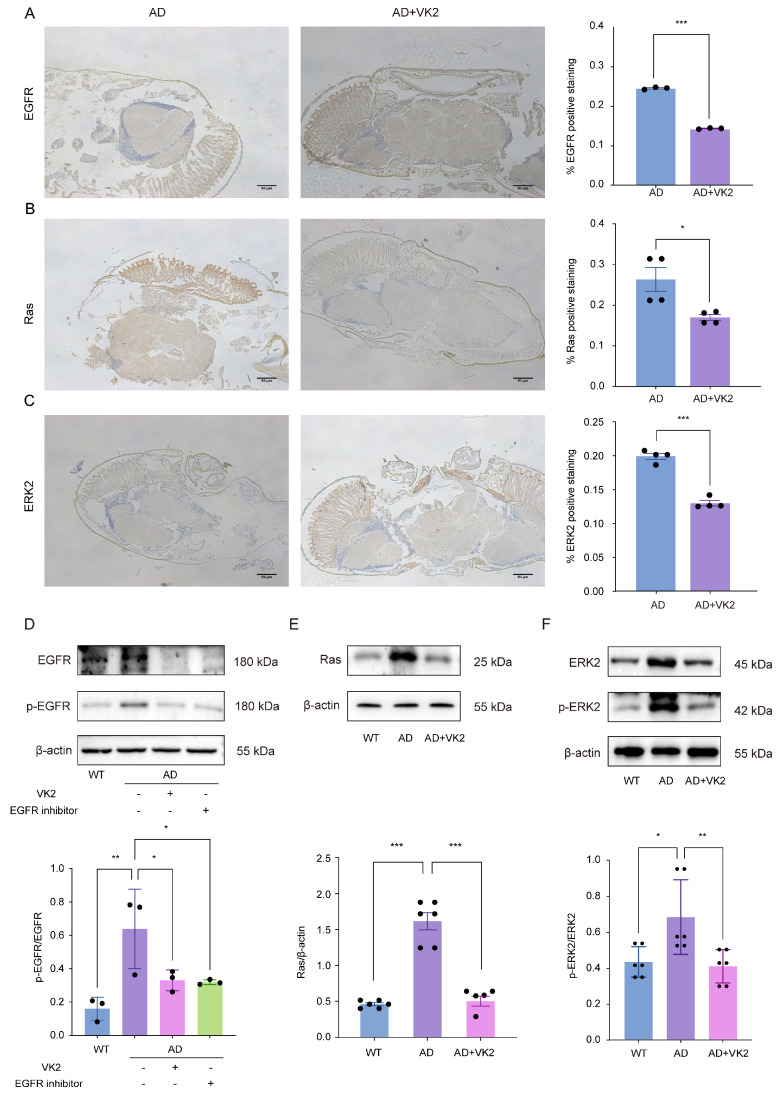
The Effect of Vitamin K2 on the protein levels of key targets. The protein levels of EGFR, Ras and ERK2 were detected by Western blot and immunohistochemistry. (**A**–**C**) Immunohistochemical staining of key target proteins (EGFR, Ras, ERK2) after VK2 treatment (**left**) and quantitative analysis results (**right**). Brown: positive protein staining, blue: hematoxylin-counterstained nuclei. Scale: 50 μm. (**D**) Western blot of EGFR protein and p-EGFR protein levels in each group (**up**), quantitative quantification of the ratio of p-EGFR to total EGFR (**down**). (**E**) Western blot diagram of Ras after VK2 treatment (**up**), quantitative diagram of Ras protein level (**down**). (**F**) Western blot of ERK2 and p-ERK2 protein levels in each group (**up**), quantitative quantification of the ratio of p-ERK2 to total ERK2 (**down**). WT group: elav-W1118; AD group: elav-Aβ42; AD + VK2 group: elav-Aβ42 + 0.5 mM VK2; AD + EGFR inhibitor: elav-Aβ42 + 100 μg/mL Gefitinib. Data information: Data are presented as mean ± standard error of mean (SEM). Panel (**A**–**C**) was analyzed by *t*-test (*n* = 5 biological replicates per group), Panels (**D**–**F**) by One-Way ANOVA followed by Tukey’s post hoc test (*n* = 50 biological replicates per group). Statistical annotation: * *p* < 0.05; ** *p* < 0.01; *** *p* < 0.001.

To determine if these transcriptional changes corresponded to differences in protein abundance and localization, we performed immunohistochemistry (IHC) on brain sections. AD model flies displayed dense and hypertrophic immunoreactive clusters of EGFR, Ras and ERK2 proteins, reflecting a pathologically high protein load induced by Aβ42. Following 0.5 mM VK2 treatment, the intensity of these immunoreactive signals was visibly reduced (Figure 8A–C). Quantitative IHC analysis confirmed that EGFR protein levels decreased from 0.24 ± 0.01 to 0.14 ± 0.01 units (*n* = 5 brains per group, *p* < 0.001), while Ras and ERK2 protein levels also showed significant reductions (*p* < 0.01). This reduction in protein accumulation aligns with the downregulation observed in the mRNA assays and confirms the efficacy of VK2 in reducing signaling protein density.

Given the predicted role of EGFR as the primary molecular target, we assessed its phosphorylation state using the clinical EGFR inhibitor Gefitinib as a reference standard to confirm target specificity. Western blot analysis on brain protein extracts demonstrated that the ratio of phosphorylated EGFR to total EGFR was significantly increased in the AD group (0.64 ± 0.24). Both 0.5 mM VK2 and Gefitinib effectively suppressed this activation, with VK2 reducing the ratio to 0.33 ± 0.06 (Figure 8D). The comparable inhibitory potency of VK2 and Gefitinib provides robust evidence that EGFR serves as a central target of VK2 action. Correspondingly, VK2 intervention significantly reduced total Ras protein levels (0.52 ± 0.17 vs. 1.66 ± 0.30 in AD; *n* = 45, *p* < 0.001) and the ratio of phosphorylated ERK2 to total ERK2 (0.41 ± 0.09 vs. 0.68 ± 0.21 in AD; *p* < 0.01; Figure 8E,F). Collectively, these findings establish that VK2 mitigates AD-associated pathologies by quenching the overactivation of the EGFR-Ras-ERK signaling axis, thereby preserving mitochondrial integrity and locomotor function.

## 3. Discussion

The findings of this study provide a comprehensive link between the neuroprotective effects of VK2 and the regulation of mitochondrial homeostasis via the EGFR-Ras-ERK signaling pathway in an AD model. Our results demonstrate that VK2 intervention effectively rescues the characteristic locomotor decline of Aβ42-expressing *Drosophila*, as evidenced by the restoration of both vertical negative geotaxis and spontaneous planar locomotion (Figure 1). Since progressive motor coordination loss is a key indicator of neurodegenerative advancement in animal models [19], the behavioral recovery observed at the 25-day mark suggests that VK2 provides a sustained functional buffer against Aβ42-induced neurotoxicity. This phenotypic improvement was accompanied by a reduction in Aβ42 protein levels in brain tissues (Figure 7A), suggesting that VK2 treatment may influence Aβ42 burden in the fly brain. However, the current study does not determine whether this effect reflects direct modulation of Aβ42 production or clearance, or whether it occurs secondarily to improvements in mitochondrial homeostasis and EGFR-Ras-ERK signaling regulation.

The behavioral improvements observed in our study are fundamentally supported by the restoration of mitochondrial ultrastructural integrity in the fly brain. Mitochondrial fragmentation and swelling are hallmark pathologies in AD neurons, directly impairing the physical scaffolding required for cellular respiration [20]. Unlike prior investigations that focused on peripheral muscle tissues, our direct TEM imaging of brain sections confirmed that VK2 intervention prevents the dissolution of mitochondrial cristae and reduces the proportion of degenerated organelles by over 50.0% (Figure 2A,B). By preserving the internal architecture of mitochondria, VK2 ensures the maintenance of the electrochemical potential across the inner membrane. This maintenance is essential for preventing the initiation of apoptotic signaling cascades in the central nervous system (CNS) [21].

This structural preservation directly translates into the revitalization of mitochondrial bioenergetic capacity and energy homeostasis. Neuronal cells account for a disproportionate amount of the total energy demand of the brain, a process that relies almost exclusively on oxidative phosphorylation (OXPHOS) [22]. During OXPHOS, substrates such as NADH provide electrons that flow through the electron transport chain to drive the synthesis of ATP from ADP [23]. We observed that the significant ATP depletion and the reduced NAD^+^/NADH ratio in AD model flies were robustly reversed by VK2 intervention (Figure 2E–G). These results align with previous hypotheses that VK2 (menaquinone) functions as an electron carrier similar to Coenzyme Q, thereby maintaining electron transfer flow and quenching excessive ROS production [24]. By stabilizing the mitochondrial membrane potential (MMP) and restoring energy output [25], VK2 effectively prevents the metabolic exhaustion that leads to neuronal death.

Beyond its role in organelle stabilization, this study prioritizes the EGFR-Ras-ERK signaling axis as a candidate pathway potentially associated with VK2-mediated neuroprotective effects. The selection of this specific pathway was not guided by an arbitrary preference but was dictated by an unbiased integration of network pharmacology and human clinical transcriptomic analysis. Specifically, weighted gene co-expression network analysis (WGCNA) identified the EGFR-related signaling cascade as the hub module with the most robust correlation to AD diagnosis in the GSE48350 dataset. While EGFR is recognized for its pleiotropic roles in normal neural development, its pathological overactivation in the context of Aβ42 toxicity creates a distinct therapeutic window. Network pharmacology and molecular docking analyses identified the EGFR-Ras-ERK axis as a candidate signaling hub potentially associated with VK2 activity. Gefitinib was included as a reference EGFR inhibitor in downstream validation experiments, and VK2 exhibited docking affinities comparable to those of the clinical inhibitor Gefitinib (Figure 3, Figure 4, Figure 5 and Figure 6). Furthermore, molecular dynamics simulation demonstrated that the VK2–EGFR complex remained structurally stable throughout the simulation period, while MM-GBSA analysis supported the energetic plausibility of the interaction. Key residues including VAL702, LEU820, and CYS773 contributed substantially to the predicted binding stability, further supporting the possibility that VK2 may interact with EGFR-associated signaling components under dynamic conditions (Figure 6). In vivo experiments in *Drosophila* brain tissues showed that VK2 was associated with reduced activation of this signaling pathway at both the transcriptional and protein levels (Figure 7). Previous reports have demonstrated that excess EGFR enhances memory loss in AD models, and inhibiting EGFR phosphorylation provides neuroprotection in other neurodegenerative contexts such as Parkinson’s disease [26,27]. Our findings show that VK2 treatment is associated with reduced EGFR phosphorylation, suggesting that it may serve as a potentially lower-toxicity modulator to clinical EGFR inhibitors for maintaining neural signaling balance in the central nervous system (Figure 8D).

A significant contribution of our work is the elucidation of the crosstalk between EGFR-Ras-ERK signaling and mitochondrial stability. While previous studies identified VK2 as an electron carrier in Parkinson’s disease models [28], our findings suggest a potential association between VK2-mediated preservation of mitochondrial cristae and modulation of the EGFR-Ras-ERK signaling axis in the context of AD. These findings suggest a potential link between signaling dysregulation and mitochondrial energetic dysfunction. EGFR is localized to mitochondria, where its signaling can promote mitochondrial biosynthesis and TCA cycle flux [29]. Furthermore, Ras proteins are major components of signaling pathways that integrate metabolic reprogramming with cellular survival, and the reduced Ras signaling observed following VK2 treatment aligns with recent post-mortem human brain studies identifying the Ras-MAPK pathway as an early driver of AD pathology [30,31]. We observed that reduced ERK2 phosphorylation following VK2 treatment was associated with improved mitochondrial integrity, as ERK2 signaling regulates the nuclear expression of mitochondrial proteins and is necessary for PINK1 Parkin-mediated mitophagy [32,33]. The suppression of Ras/ERK overactivation observed following VK2 treatment likely stabilizes the NADH redox state and prevents the signaling-induced mitochondrial decay that otherwise characterizes the AD brain [34].

Although this study provides relatively systematic evidence from transcriptomic analysis, molecular docking, and in vivo experiments, several limitations should be acknowledged. (1) At the mechanistic level, while the existing data support an association between VK2 and reduced EGFR-Ras-ERK pathway activity, a direct target-binding interaction and definitive mechanistic causality have not been established; kinase activity assays and pathway rescue experiments are still required to exclude the possibility of indirect effects. (2) At the model level, this study was conducted primarily using an Aβ42-induction model, and the findings should therefore be interpreted within the context of amyloid-centered AD pathology. Whether VK2 modulates tau phosphorylation, aberrant aggregation, and tau-mediated neurodegeneration remains to be investigated, and systematic assessment of synaptic protein expression and neuronal connectivity—both of which are hallmarks of early AD pathology—was not performed. (3) At the translational level, although the *Drosophila* model offers well-recognized advantages in genetic manipulation and high-throughput screening, it cannot fully recapitulate the complex pathological microenvironment of the human CNS (Central Nervous System, CNS), and the absence of parallel mammalian model validation limits the broader applicability of the current findings. Notably, prior studies have reported that menaquinones exhibit prolonged systemic circulation and preferential accumulation in brain tissue, and ADMET predictions further suggest favorable intestinal absorption, moderate blood–brain barrier permeability, and high lipophilicity for VK2, collectively implying reasonable CNS exposure potential. Nevertheless, the actual pharmacokinetic profile and long-term safety of VK2 in humans remain to be systematically characterized. Taken together, these limitations highlight the need for future studies to deepen mechanistic elucidation, expand pathological model coverage, and advance mammalian in vivo validation to strengthen the reliability and clinical translational value of the present findings.

**Figure 9:**
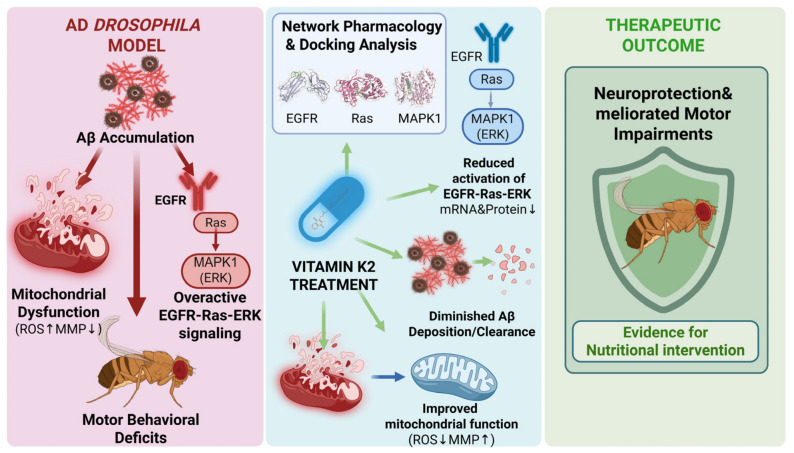
Schematic diagram illustrating the overall workflow of this study.

In summary, this study demonstrates that VK2 is a promising nutritional intervention that mitigates AD pathology by targeting the restoration of mitochondrial homeostasis via the EGFR-Ras-ERK signaling axis (Figure 9). This molecular target was objectively prioritized through clinical data integration and validated as a functional skeleton for systematic neuroprotection. VK2 intervention achieves a systematic improvement by restoring locomotor ability, stabilizing mitochondrial membrane potential, and maintaining ATP synthesis, while inhibiting oxidative stress. Crucially, these benefits are amplified through the downregulation of the EGFR-Ras-ERK pathway, which reduces Aβ42 protein and Aβ neurotoxicity. While the reliance on the *Drosophila* model is a limitation, the high conservation of these pathways provides a strong foundation for future mammalian studies. Improved mitochondrial structural and functional integrity observed after VK2 treatment was associated with modulation of the EGFR-Ras-ERK axis. However, direct target engagement and pathway causality require further validation in future studies.

## 4. Materials and Methods

### 4.1. Materials

Vitamin K2 (MK-4, ≥98%, Cat# V863481-5g) was purchased from Macklin (Shanghai, China). Gefitinib was obtained from MedChemExpress (Shanghai, China, Cat# HY-50895, 100 mg). The Animal Tissue/Cell/Blood Genomic DNA Rapid Extraction Kit was obtained from Zhuangmeng (Beijing, China, Cat# ZP332-2). For cell-related and biochemical detection kits: the CellTiter-Lumi™ II Luminescent Cell Viability Assay Kit (Shanghai, China, Cat# C0056S), BCA Protein Detection Kit (Cat# P0010S) were all purchased from Beyotime (Shanghai, China). NAD/NADH detection kit (Suzhou, China, Cat# N6035S) was obtained from UElandy. For qPCR-related reagents: the Evo M-MLV RT Premix for qPCR Reverse Transcription Kit (Cat# AG11706) and SYBR^®^ Green Premix Pro Taq HS qPCR Kit (Cat# AG11701) were both supplied by Aikerkai (Changsha, China).

Additionally, tetramethylrhodamine ethyl ester (TMRE) dyes (Cat# HY-D0985A) and H_2_DCFDA probe (Cat# HY-D0940) were purchased from MCE (Shanghai, China). Color PAGE Gel Rapid Preparation Kit (Cat# PG112); SuperKine™ West Femto Maximum Sensitivity Substrate (Cat# BMU102) was obtained from Epizym (Shanghai, China). Amyloid β1-42 (Aβ1-42) ELISA Kit (Cat# NL-5481) was purchased from NaLian (Shanghai, China). Anti-EGFR Rabbit antibody (Cat# R22778, RRID: AB_3719349), Anti-ERK2 Rabbit antibody (Cat# 343830, RRID: AB_3073887), Anti-Ras Rabbit antibody (Cat# CY5223, RRID: AB_3719348), Anti-Phospho-EGFR Rabbit antibody (Cat# R24173, RRID: AB_10588471), Anti-Phospho-ERK2 Rabbit antibody (Cat# 310065, RRID: AB_3719029) was obtained from Zenbio (Chengdu, China), Anti-β-actin Rat antibody (Cat# R380624, RRID: AB_3719351), Goat anti-rabbit IgG (HRP) secondary antibody (Cat# MK103, RRID: AB_3719379), Goat anti-Rat IgG (HRP) secondary antibody (Cat# MK102A, RRID: AB_3719380) was obtained from BIOMIKY (Shanghai, China).

### 4.2. Fly Stocks

The fly strains utilized in this study are as follows: wild-type W1118 (served as the control), UAS-Aβ42 (expressing the human Aβ42 gene downstream of the UAS promoter), and elav-GAL4 (a pan-neuronal-specific GAL4 driver line). The W1118 and elav-GAL4 strains were generously provided by Professor Tian Wei from Anhui Medical University, while the UAS-Aβ42 strain was kindly gifted by Professor Guojin Cao from Gannan Medical University.

### 4.3. Experimental Groups

Virgin females of the elav-GAL4 strain were crossed with males of the UAS-Aβ42 strain. To avoid parental interference, all parental flies were removed on the 5th day post-hybridization. After the F1 eggs hatched and developed into adult flies, male offspring were collected as the AD transgenic *Drosophila* model—this model specifically overexpresses the human Aβ42 gene in the nervous system, with the genotype Aβ42/y; elav/+. Designated as elav/Aβ42, this transgenic strain harbors an overexpressed human Aβ42 gene sequence in its nervous system. Virgin females of the elav-GAL4 strain were crossed with males of the W1118 strain. To avoid parental interference, all parental flies were removed on the 5th day post-hybridization. After the F1 eggs hatched and developed into adult flies, male offspring were collected as the WT group.

After the experiment, the following groups were used: (1) WT group: elav-W1118 group, (2) AD group: elav-Aβ42 group, (3) AD + 0.1 mM VK2 group: elav-Aβ42 + 0.1 mM Vitamin K2 group, (4) AD + 0.5 mM VK2 group: elav-Aβ42 + 0.5 mM Vitamin K2 group, (5) AD + 1 mM VK2 group: elav-Aβ42 + 1 mM Vitamin K2 group, (6) AD +100 μg/mL Gefitinib group. Subsequently, the optimal concentration and treatment time were determined according to *Drosophila* behavioral tests, including climbing assays.

The intervention protocol for Vitamin K2 was as follows: Vitamin K2 powder was dissolved in anhydrous ethanol to prepare a 0.1 M stock solution, which was stored at −20 °C. After the stock solution was fully dissolved at room temperature, intervention systems with final concentrations of 0.1 mM, 0.5 mM, and 1 mM VK2 were prepared. For administration: *Drosophila* were intervened with medium containing VK2 starting from the first day post-eclosion. Meanwhile, the normal control group (WT group) and AD model group were given an equal volume of vehicle, which was identical to the VK2-containing medium except for the absence of VK2.

According to the literature [35], 100 mg Gefitinib stock liquor was configured into a Gefitinib solution with a final concentration of 100 μg/mL in a common medium, and the intervention time was 25 days. From the first day after the emergence of *Drosophila*, the medium containing Gefitinib was used for intervention. At the same time, the normal control group (WT group) and the AD model group were given an equal volume of solvent, except for the absence of Gefitinib; the rest were the same as the medium containing Gefitinib.

### 4.4. Walking Assay

Adult flies subjected to 25 days of intervention were gently transferred to a circular plastic chamber (35 mm × 3.5 mm) as the bioassay platform, followed by a 10 min acclimation period prior to the initiation of measurements. Subsequently, the bioassay area was video-recorded for 6 min. The animal behavior analysis software AnimalTA 3.2.2 version was used to track the movement trajectories, measure the walking distance, and calculate the walking speed of the flies, so as to evaluate their motor activity.

### 4.5. Climbing Assay

Flies were anesthetized with CO_2_, and 10 flies were rapidly transferred to each transparent glass tube (length: 23 cm, diameter: 2.5 cm). Each group was set with at least six replicates, and eight replicates were used in this experiment. A marking line was drawn 6 cm above the bottom of each glass tube. After the flies recovered from CO_2_ anesthesia for 30–60 min, the glass tube was gently tapped to allow all flies to land at the bottom. Once all flies had settled at the bottom, the number of flies that climbed above the 6 cm marking line within 10 s was recorded.

The Climbing Index (CI) was calculated using the formula: CI = A/Total, where A represents the number of flies that climbed above the marking line, and Total is the total number of flies per tube. Each tube was tested at least three times.

### 4.6. Network Pharmacological Analysis

Acquisition of VK2-related information and potential target genes. The 3D structure and SMILES of VK2 were obtained by searching the PubChem database: https://pubchem.ncbi.nlm.nih.gov/ (accessed on 10 April 2025) [36], Subsequently, the SMILES of VK2 was input into three databases, including PharmMapper [37] https://www.lilab-ecust.cn/pharmmapper/ (accessed on 10 April 2025), TargetNet http://targetnet.scbdd.com/ (accessed on 10 April 2025) database and Swiss Target Prediction database http://www.swisstargetprediction.ch (accessed on 10 April 2025) to screen out 746 potential target genes of VK2. All screened genes were further analyzed by Molecular Complex Detection (MCODE). MCODE analysis was performed to identify densely connected modules within the protein–protein interaction (PPI) network. The top enriched KEGG pathways for each module are shown.

Retrieval of AD-related datasets and analysis of differentially expressed genes (DEGs). The GSE48350 dataset was retrieved from the Gene Expression Omnibus (GEO) database http://www.ncbi.nlm.nih.gov/geo (accessed on 20 April 2025). Differential expression analysis was first performed to identify DEGs, followed by Gene Set Enrichment Analysis (GSEA), and proteins associated with the most relevant AD-related enriched pathways were obtained. Weighted Gene Co-expression Network Analysis (WGCNA) was applied to analyze DEGs between AD patients and normal controls. Three modules (MEpeachpuff3, MElightblue1 and MEsnow) were selected as the key gene modules of AD according to the principle of the highest correlation coefficient and the strongest significance (minimum *p* value). The key module genes identified by WGCNA were intersected with genes retrieved from the Online Mendelian Inheritance in Man (OMIM), GeneCards, and DisGeNET databases. AD-related genes were retrieved from GeneCards, DisGeNET, and OMIM using the keyword “Alzheimer’s disease”. Genes with a GeneCards relevance score ≥ 10 and a DisGeNET GDA score ≥ 0.1 were retained. For OMIM, all retrieved disease-associated genes were collected and deduplicated, with priority given to Gene Map entries with phenotype mapping key = 3. Venn diagrams were generated using the online tool VENNY 2.1.27 https://bioinfogp.cnb.csic.es/tools/venny/index.html (accessed on 15 May 2025) to visualize the intersection results.

Construction of protein–protein interaction (PPI) network and enrichment analysis. Intersection analysis was conducted between VK2 potential target genes and AD-related key gene clusters to obtain shared protein targets of VK2 and AD. These shared targets were uploaded to the STRING database https://cn.string-db.org/ (accessed on 20 May 2025) for PPI network construction, and the downloaded files were imported into Cytoscape 3.9.1 software [38] for visualization. Additionally, Gene Ontology (GO) and Kyoto Encyclopedia of Genes and Genomes (KEGG) enrichment analyses were performed on the potential targets of VK2 in AD treatment using R language (RStudio 2024.12.0 version), and the results were visualized.

### 4.7. Molecular Docking of VK2 with the Target Protein

The two-dimensional structures of VK2 and gefitinib were downloaded from the PubChem database https://pubchem.ncbi.nlm.nih.gov/ (accessed on 10 April 2025). The PDB files of the key targets EGFR (PDB ID: 3POZ), ERK (PDB ID: 5ETC) and Ras (PDB ID: 5WDS) obtained by the method under Part 6 were downloaded from the protein database https://www.rcsb.org/ (accessed on 13 June 2025). Molecular docking was performed using the Sail Vina platform. OpenBabel and PyMOL 3.1.0 version software were used to visualize the molecular docking results, and then the PLIP website https://projects.biotec.tu-dresden.de/plip-web/plip/index (accessed on 13 June 2025) was used to determine the binding site.

### 4.8. Molecular Dynamics

A 200 ns molecular dynamics (MD) simulation of the protein–ligand complex was performed using Gromacs 2025. The AMBER14SB and GAFF2 force fields were applied for the protein and ligand, respectively. The complex was placed in a cubic box under periodic boundary conditions and solvated with the TIP3P water model. Na^+^/Cl^−^ ions were added to neutralize the system and maintain a physiological ionic concentration of 0.15 M. Following energy minimization, NVT and NPT equilibration were performed prior to the 200 ns production simulation at 310 K and 1 bar with a 2 fs timestep. Binding free energies of the stabilized complex were subsequently calculated using the MM-PBSA method.

### 4.9. Transmission Electron Microscopy

*Drosophila* brain tissue samples were fixed in 2.5% glutaraldehyde (MCE, Shanghai, China, GA; Electron Microscopy Sciences) at room temperature for 2–3 h. After rinsing with PBS, the samples were further fixed in 1% osmium tetroxide (OsO_4_) buffer for 2 h, followed by dehydration through a graded ethanol series. Subsequently, the samples were embedded in Epon812 resin (SPI Supplies, West Chester, PA, USA). Ultrathin sections (70 nm) were stained with 2% uranyl acetate for 30 min and then with lead citrate for 10 min. Finally, the sections were imaged using a transmission electron microscope (Tecnai™ Spirit; FEI, Chinese Academy of Sciences, Beijing, China).

### 4.10. Mitochondrial Membrane Potential (MMP) Assay

Mitochondrial membrane potential was evaluated in freshly dissected adult fly brains by TMRE staining under live-tissue conditions. Briefly, brains from 5 to 10 flies per group were dissected in ice-cold 1× PBS and incubated with TMRE (1 μM) for 15 min at room temperature in the dark. After staining, samples were washed three times with dye-free buffer and mounted in fresh buffer for immediate imaging. All images were acquired using identical exposure settings. Fluorescence intensity was quantified with ImageJ 1.62 version in the same brain region for each sample after background subtraction, and the average TMRE signal was used as a readout of mitochondrial membrane potential.

### 4.11. ROS Assay

Intracellular ROS levels were assessed in freshly dissected, unfixed adult *Drosophila* brains using the cell-permeable fluorescent probe H_2_DCFDA under live-tissue conditions. Briefly, brains from 5 to 10 flies per group were rapidly dissected in ice-cold 1× PBS and immediately incubated with H_2_DCFDA working solution (final concentration: 10 μM) for 20 min at room temperature in the dark. After staining, the samples were gently washed three times with dye-free 1× PBS and mounted in fresh buffer for immediate imaging. Fluorescence images were acquired using identical microscope settings for all groups under the FITC channel (Ex/Em = 488/525 nm). The fluorescence intensity of H_2_DCFDA was quantified in the same anatomical region of each brain using ImageJ 1.62 version after background subtraction, and the mean fluorescence intensity was used as an index of intracellular ROS levels.

### 4.12. ATP Assay

According to the instructions of the CellTiter-Lumi™ II cell viability assay kit, heads from 30 to 40 flies per group were collected for ATP extraction from elav-Aβ42 male *Drosophila melanogaster* treated or untreated with VK2. After quantification, 100 μL of protein sample was added with 100 μL of ATP luminescence detection solution to a black 96-well plate and placed in a microplate reader for luminescence detection.

### 4.13. NAD^+^/NADH Assay

The NAD^+^/NADH index was determined according to the requirements of the instructions in the NAD^+^/NADH detection kit. Each group was prepared to dissect 40 *Drosophila* heads, homogenize for 10 min, 12,000× *g*, centrifuge at 4 °C for 10 min, take the supernatant (for later use), prepare 10 mM NADH standard, dilute the standard, and prepare ethanol dehydrogenase working solution. The total amount of NAD^+^/NADH, the specific contents of NAD^+^ and NADH, and the NAD^+^/NADH ratio in the samples were quantified according to the manufacturer’s protocol. Finally, the sample was added to a 96-well plate according to the instructions, and the alcohol dehydrogenase working solution was slowly added and fully mixed. Each well was added with 10 μL color solution, fully mixed and incubated at room temperature for 30 min, and the absorbance at 450 nm was measured for data analysis.

### 4.14. ELISA Assay

The level of Aβ42 protein in the brain tissue of each group of *Drosophila* was measured according to the Human β-amyloid protein 1–42 (Aβ1–42) ELISA detection kit (Shanghai, China, NaLian), and the expression level of Aβ1–42 in the brain of AD *Drosophila* after VK2 treatment was evaluated.

### 4.15. RT-qPCR

After 25 days of VK2 administration, the reverse transcription reaction system was prepared according to the instructions of Evo M-MLV RT Premix for qPCR reverse transcription kit, and the RT-qPCR reaction system was prepared according to the instructions of SYBR Green kit for amplification. The primers listed in Table 1 were obtained from the PrimerBank database and synthesized by Anhui General Biotechnology Co., Ltd. (Chuzhou, China).

### 4.16. Immunohistochemistry

The heads of *Drosophila melanogaster* in each group were fixed overnight in 4% paraformaldehyde, dehydrated by ethanol gradient, embedded in paraffin, and prepared into 5 μm thick serial sections. After xylene dewaxing and gradient ethanol hydration, the sections were placed in citrate buffer (pH 6.0) for antigen heat repair. Endogenous peroxidase activity was blocked with 3% H_2_O_2_ and blocked with 5% goat serum blocking solution at room temperature for 1 h. Subsequently, primary antibodies: Anti-EGFR Rabbit antibody (1:1000), Anti-ERK Rabbit antibody (1:1000), Anti-Ras Rabbit antibody (1:1000) were added dropwise and incubated overnight at 4 °C. After washing with PBS the next day, HRP-labeled secondary antibody was added and incubated at room temperature for 1 h. The DAB kit was used for color development, and the nucleus was re-stained with hematoxylin. Finally, after dehydration, transparency and sealing, the images were observed and collected under an optical microscope. Using ImageJ 1.62 version software, five visual fields were randomly selected in the same brain region of each sample, and the integrated optical density (IOD) of positive staining was measured and calculated for semi-quantitative analysis.

### 4.17. Western Blot

Collected *Drosophila* head samples were homogenized in ice-cold RIPA lysis buffer supplemented with protease and phosphatase inhibitor cocktails. Samples were then thoroughly homogenized by ultrasonic treatment. The protein concentration of the lysate was determined using a BCA Protein Assay Kit. After heating the samples at 100 °C for 10 min to denature proteins, 5 μg of total protein per sample was loaded onto SDS-PAGE gels for separation. The separated proteins were transferred to a membrane, which was then incubated with the indicated primary antibodies diluted in primary antibody diluent at 4 °C overnight. Subsequently, the membrane was incubated with secondary antibodies, and immunoblot signals were detected using an ECL substrate. Signal quantification was performed using ImageJ 1.62 version software. The antibodies used are listed as follows: Anti-EGFR Rabbit antibody (1:1000), Anti-ERK Rabbit antibody (1:1000), Anti-Ras Rabbit antibody (1:1000), Anti-Phospho-EGFR Rabbit antibody (1:1000), Anti-Phospho-ERK Rabbit antibody (1:1000), Anti-β-actin Rat antibody (1:6000), Goat anti-rabbit IgG (HRP) secondary antibody (1:20,000), Goat anti-Rat IgG (HRP) secondary antibody (1:15,000).

### 4.18. Data Analysis

All experimental data were statistically analyzed and graphed using GraphPad Prism 9.5.3 software, with results expressed as mean ± standard error of mean (SEM). One-way analysis of variance (ANOVA) or two-way ANOVA was used for comparisons among multiple groups. If the ANOVA results were statistically significant, Tukey’s post hoc test was performed for pairwise comparisons. mRNA levels were analyzed using Roche LightCycler 96 1.1.0.1320 version data processing software, and differences between samples were tested by one-way ANOVA. A *p*-value < 0.05 was considered statistically significant in all experiments. Adobe Illustrator 2020 software was used for figure assembly and optimization. The statistical significance of enrichment analysis was corrected by Benjamini–Hochberg false discovery rate (FDR), and the threshold was FDR < 0.05.

## 5. Conclusions

In conclusion, this study demonstrates that VK2 mitigates AD pathology by preserving mitochondrial integrity and modulating the EGFR-Ras-ERK signaling pathway. In an AD *Drosophila* model, VK2 intervention restores locomotor function and protects mitochondrial ultrastructure by preventing cristae dissolution. These neuroprotective benefits are primarily associated with the downregulation of the EGFR-Ras-ERK signaling axis, which was accompanied by reduced Aβ42 protein levels and attenuated neurotoxicity. By linking signaling pathway regulation to organelle repair, our findings establish VK2 as a promising therapeutic candidate and support its further evaluation in mammalian models.

## Figures and Tables

**Table 1:** Primer sequence of RT-qPCR.

Gene	Primer Sequence (5′−3′)
EGFR (F)	AGGGCATGTCGTATCTGGAG
EGFR (R)	GCCCAAAGTCGGTGATCTTC
ERK (F)	GCTGAAGTGCCATTTCGGAT
ERK (R)	TAAGGCGCATTGTCTGGTTG
Ras (F)	GGCGAGTACTTCTTCTCCGA
Ras (R)	GTAGATAGCGCAGCACACTG

## Data Availability

The raw data supporting the conclusions of this article will be made available by the authors on request.

## Supplementary Materials

The following supporting information can be downloaded at: https://www.mdpi.com/article/10.3390/ijms27135708/s1.

## Abbreviations

The following abbreviations are used in this manuscript:

AD	Alzheimer’s Disease
Aβ	β-amyloid
VK2	Vitamin K2
GO	Gene Ontology
KEGG	Kyoto Encyclopedia of Genes and Genomes
TEM	Transmission electron microscopy
MMP	Mitochondrial membrane potential
ROS	Oxygen species
EGFR	Epidermal Growth Factor Receptor
Ras	Rat Sarcoma oncogene
ERK	Extracellular Signal-Regulated Kinase
MQC	Mitochondrial quality control
PPI	Protein–protein interaction
MCODE	Molecular Complex Detection
WGCNA	Weighted Gene Co-expression Network Analysis
CNS	central nervous system
OXPHOS	oxidative phosphorylation
OsO_4_	osmium tetroxide
