# Supplementary material for: Vitamin K2 Promotes Mitochondrial Structural and Functional Homeostasis to Ameliorate Alzheimer Pathology by Targeting the EGFR-Ras-ERK Signaling Axis
Source: Int J Mol Sci. 2026 Jun 24;27(13):5708. doi: 10.3390/ijms27135708 (PMC13361347; doi:10.3390/ijms27135708)
Supplement: Supplementary file 1 [file ijms-27-05708-s001.zip › ijms-4321965-supplementary.pdf]

Supplementary Figure

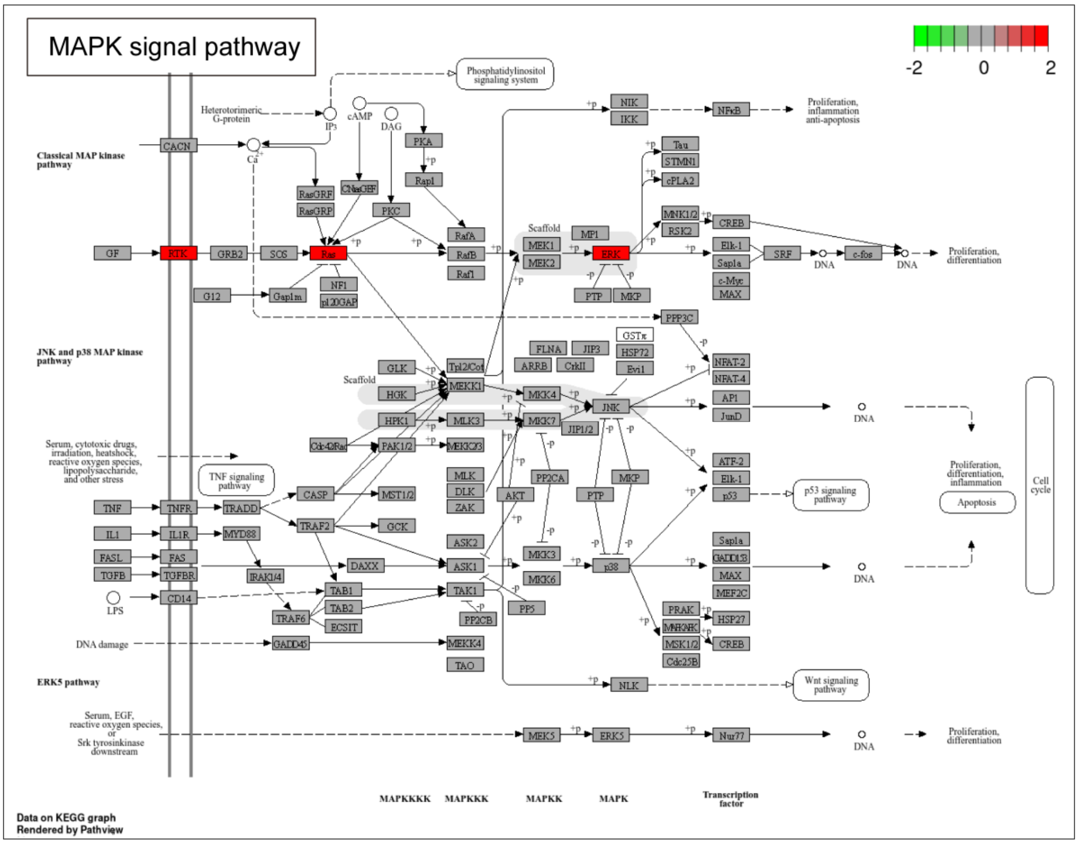

Figure S1. MAPK pathway map. Pathway map illustrating the ERK signaling cascade: growth factors (e.g., EGF, PDGF) activate RTKs, which signal through Ras–Raf–MEK1/2–ERK1/2 to regulate proliferation, differentiation, and anti-apoptosis. Color bar: -log10(P value); which warmer colors denote higher significance.
